# The Visual Dictionary of Antimicrobial Stewardship, Infection Control, and Institutional Surveillance Data

**DOI:** 10.3389/fmicb.2021.743939

**Published:** 2021-10-28

**Authors:** Julia Keizer, Christian F. Luz, Bhanu Sinha, Lisette van Gemert-Pijnen, Casper Albers, Nienke Beerlage-de Jong, Corinna Glasner

**Affiliations:** ^1^Centre for eHealth and Wellbeing Research, Section of Psychology, Health and Technology, University of Twente, Enschede, Netherlands; ^2^Department of Medical Microbiology and Infection Control, University of Groningen, University Medical Center Groningen, Groningen, Netherlands; ^3^Heymans Institute for Psychological Research, University of Groningen, Groningen, Netherlands; ^4^Technical Medical Center, Section of Health Technology and Services Research, University of Twente, Enschede, Netherlands

**Keywords:** data visualization, visual dictionary, antimicrobial stewardship, infection control, surveillance

## Abstract

**Objectives:** Data and data visualization are integral parts of (clinical) decision-making in general and stewardship (antimicrobial stewardship, infection control, and institutional surveillance) in particular. However, systematic research on the use of data visualization in stewardship is lacking. This study aimed at filling this gap by creating a visual dictionary of stewardship through an assessment of data visualization (i.e., graphical representation of quantitative information) in stewardship research.

**Methods:** A random sample of 150 data visualizations from published research articles on stewardship were assessed (excluding geographical maps and flowcharts). The visualization vocabulary (content) and design space (design elements) were combined to create a visual dictionary. Additionally, visualization errors, chart junk, and quality were assessed to identify problems in current visualizations and to provide improvement recommendations.

**Results:** Despite a heterogeneous use of data visualization, distinct combinations of graphical elements to reflect stewardship data were identified. In general, bar (*n* = 54; 36.0%) and line charts (*n* = 42; 28.1%) were preferred visualization types. Visualization problems comprised color scheme mismatches, double *y*-axis, hidden data points through overlaps, and chart junk. Recommendations were derived that can help to clarify visual communication, improve color use for grouping/stratifying, improve the display of magnitude, and match visualizations to scientific standards.

**Conclusion:** Results of this study can be used to guide data visualization creators in designing visualizations that fit the data and visual habits of the stewardship target audience. Additionally, the results can provide the basis to further expand the visual dictionary of stewardship toward more effective visualizations that improve data insights, knowledge, and clinical decision-making.

## Introduction

The amount of and reliance on data increases with the increase of scientific publications and information technologies in healthcare (Murdoch and Detsky, 2013; Wang et al., 2018). These big data raise various issues to be resolved by innovative big data analytics, including integrating, analyzing, and visualizing data to translate them into meaningful information (Khan et al., 2014; Ambigavathi and Sridharan, 2018). The translation and communication to specific target groups is challenging (Murdoch and Detsky, 2013). Without this translation and communication, researchers and practitioners cannot optimally use the information, so that the true value of the data remains hidden. Data visualization, here defined as the graphical representation of quantitative information, can facilitate the transformation of data to understandable and actionable information and improve memorization and communication. Data visualization also aids in the interpretation of big data and in the understanding of sophisticated statistical models and their results – two rising trends over the last decades (Bailly et al., 2018; Adam Bohr, 2020). The importance of data visualization can, once again, be observed in the COVID-19 pandemic with the ubiquitous presence of charts and dashboards that aim to inform and support decision-making for a wide variety of target audiences (Comba, 2020).

**FIGURE 1:**
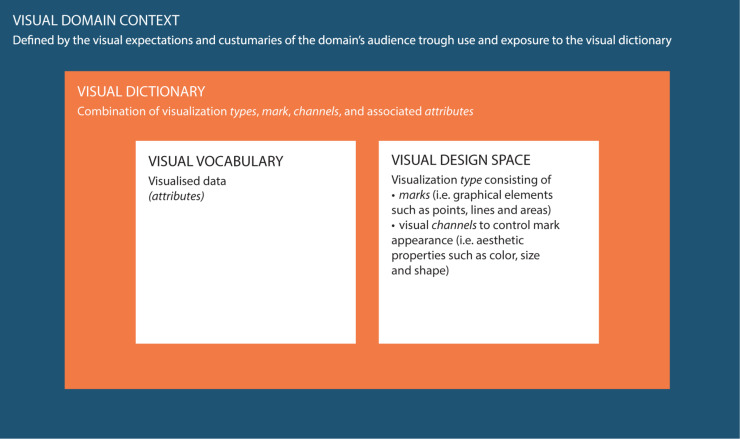
Conceptual framework used in this study to clarify the definitions and interrelations between the visual domain context, the visual dictionary and the visual domain vocabulary and visual design space. To clarify the conceptual definitions a linguistic analogy can be used: a dictionary describes language in terms of both vocabulary (i.e., the set of words familiar in a language) and grammar/punctuation (i.e., the set of structural rules and supporting marks that control the composition and navigability of sentences, phrases, and words). Similarly, the visual dictionary describes visualizations in terms of both visual vocabulary (i.e., the domain content in terms of visualized data attributes) and visual design space (i.e., graphical elements and supporting aesthetic properties). The language or visual domain context is an overarching concept that represents language/visualization in practice, i.e., expectations and customs of the target audience, and how this affects their perception and interpretation of data visualizations. The visual domain context is, just as language, subject to changes over time and subject to interpretation differences based on varying perspectives.

Data visualization is an active (research) field in itself and is generally part of statistical software for data analysis processes (e.g., R). Information on the data visualization process is numerous and can be transferred between research fields (Tufte, 2001; Kelleher and Wagener, 2011; Gatto, 2015; Evergreen, 2017). However, research on the visual domain context within a research field is often lacking, i.e., what the target audience is accustomed to see and expects in terms of content and design, and how this influences the perception and interpretation of data visualizations from different perspectives (Sedrakyan et al., 2019). Common data visualization practices in a specific domain can be identified by studying the visual dictionary, which consists of the visual vocabulary and visual design space (see Figure 1; Munzner, 2014). The vocabulary represents the content in terms of visualized data attributes. The design space is “an orthogonal combination of two aspects,” namely marks (i.e., graphical elements such as points, lines and areas) and visual channels to control their appearance (i.e., esthetic properties such as color, size and shape) (Munzner, 2014).

Data and data visualization play important parts in the field of infectious diseases and antimicrobial resistance (AMR) for the reporting on the growing burden on health and healthcare systems (OECD, 2018; World Health Organization, 2020b). Comprehensible and actionable information on antimicrobial consumption, pathogen distribution, or incidence and prevalence of (multi-) drug resistant microorganisms are vital to design interventions to tackle the AMR challenge (World Health Organization, 2015). Reliable data on AMR, robust data analyses, and the correct presentation of data are essential to support crossing borders between human, animal, and ecosystem health, also known as the One Health approach. One example is the surveillance of AMR in humans, animals, and food (European Centre for Disease Prevention and Control [ECDC], European Food Safety Authority [EFSA], and European Medicines Agency [EMA], 2017). In this study, we focus on the hospital level, where antimicrobial and diagnostic stewardship, infection control, and institutional surveillance (further summarized under “stewardship”) are the core components of strategies that promote the responsible use of antimicrobials and improve the quality and safety of patient care (Dik et al., 2016; Dyar et al., 2017). Data visualization is an integral part of these strategies, as it unveils the (local) situation and drivers of AMR, and can have a significant impact on the use of antimicrobials (Luz et al., 2019; Graber et al., 2020).

It has been shown how important it is to study data and data visualization experiences and perceptions in the medical domain and how these can influence the interpretation of data (Aung et al., 2019a,b). Identifying the key messages from a data visualization can be substantially hindered by a suboptimal visualization type. The audience’s background and its familiarity with data visualization (i.e., visual domain context) have to be considered in the design process to avoid these obstacles. Example studies that identified the visual domain context by studying the design space can be found in the field of genomic epidemiology and genomic data visualization (Crisan et al., 2019; Nusrat et al., 2019). Although some recommendations exist that are helpful for stewardship data visualization, common data visualizations practices in the field have yet to be revealed (Carroll et al., 2014; Salinas et al., 2020). The visual domain context and the use of data visualization in the field are unstudied – a systematic approach to define the design space is missing.

In this study, we aim to fill these gaps by assessing and defining the design space of data visualization in stewardship and to create a visual dictionary. The results of this study can help data visualization creators, such as AMR-/data-professionals and scientists, to anticipate the visual domain context of the target audience and link it with existing recommendations for the data visualization process. This could benefit both research and clinical decision-making in the translation and communication of data to understandable and actionable information needed to tackle the AMR challenge, thereby improving the quality and safety of health and healthcare.

### Study Data

This study succeeds a mapping study that clustered the AMR field into 88 topics (Luz et al., 2021). The map was generated by assessing the entire body of AMR literature available on PubMed between 1999 and 2018 (152,780 articles). Using a machine learning algorithm (STM), topics were identified based on the title and abstract text (Roberts et al., 2019). The present study used all articles of three of the identified topics: *stewardship* (*n* = 3,383 articles), *infection control* (*n* = 1,687 articles), and *institutional surveillance* (*n* = 2,176 articles). These three topics reflect the core components of an integrated, comprehensive stewardship concept in institutional healthcare (Dik et al., 2016).

**FIGURE 2:**
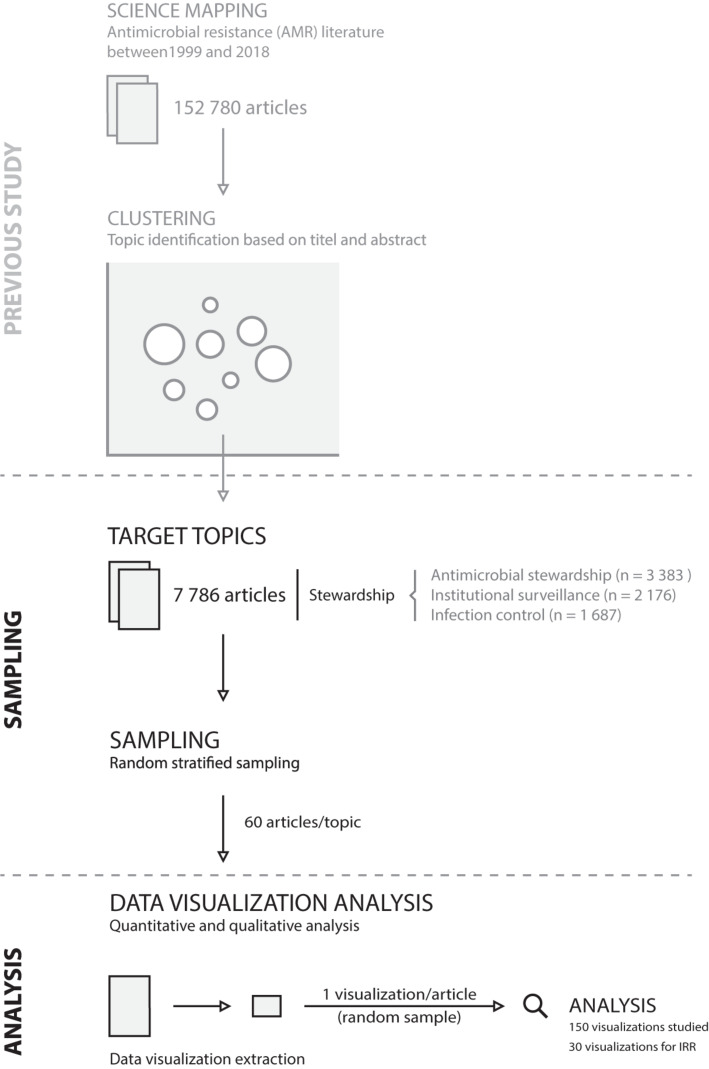
Study design. IRR, inter-rater reliability.

For each topic, a sample of 60 articles that contained at least one data visualization was randomly drawn. Data visualization was defined as the graphical representation of quantitative data. Geographical maps and flowcharts were excluded, as geographical data have distinct visual characteristics and challenges beyond the scope of this study (see e.g., Wang et al., 2017; Singleton and Arribas-Bel, 2021). From the sampled articles, one visualization per article was randomly sampled resulting in 180 data visualizations. The study design is shown in Figure 2. To analyze inter-rater reliability, 10 randomly picked data visualizations per topic were analyzed in duplicate, and the joint probability of agreement was calculated by dividing the number of agreements per categorical assessment form question (i.e., visual characteristics described in section “Data Visualization Analysis”) by the total number of assessments (O’Connor and Joffe, 2020).

### Data Visualization Analysis

The resulting 150 data visualizations (Supplementary Material S1) were analyzed using the nomenclature and categorization by Munzner adapted for this study (Munzner, 2014). This approach dissected data visualizations into visual characteristics:

- *Attributes* (*or variables*, *parameters*, *features*): the underlying data labeled as categorical, ordered, or quantitative
- *Marks*: the basic geometric element (points, lines, or areas)
- *Channel*: channels control the visual appearance of *marks*
- *Position*: horizontal, vertical, both
- *Color*
- *Shape*
- *Tilt*
- *Size*: length, area, volume

- *Channel effectiveness*
- *Magnitude*: the effectiveness to express ordered *attributes* can be ranked: position on common scale (most effective) > position on unaligned scale > length > tile/angle > area > depth > color luminance/saturation > curvature/volume (least effective)
- *Identity*: the effectiveness to express categorical *attributes* can also be ordered: color hue >shape

In addition, data visualizations were labeled with the visualization type used (e.g., bar chart, line chart, scatter plot, etc.) and the use of faceting (multiple linked visualizations in a design grid). Each visualization was assessed upon its interpretability without additional text (yes, if interpretable without additional information; partially, if a description was given in a caption; not at all, if a description was absent or only available in the article text).

Visualization quality was captured by rating the first and last impression during the analysis process on a scale form 1 (poor) to 5 (good). The choice of the visualization type given the underlying data was rated on a scale from 1 (poor) to 5 (good). In addition, free, written text was recorded to capture comments and remarks about the data visualization.

A structured assessment form (Supplementary Material S2) was developed comprising all the above-mentioned elements. The form was discussed within a multidisciplinary team of data-visualization and AMR experts. The assessment form was applied to each data visualization in a two reviewer (JK and CL) process. First, the assessment form was used for training the analysis process with 10 data visualizations not part of the final study data. Next, each reviewer analyzed 50% of the study data visualizations followed by a re-review through the other researcher. Consensus was reached through discussion if the first assessment differed.

### Quantitative Analysis

Descriptive statistics were calculated for visualization type, number of attributes, faceting, rating, and visualization type choice. *Attributes* were analyzed for pairwise co-occurrence and presented if a combination occurred more than twice in total.

### Visual Dictionary

The visual dictionary was created based on the visual vocabulary (stewardship-related content) and visual design space (characteristics used to design the visualization). The vocabulary was analyzed by identifying *attributes* and grouping the *attribute* names using inductive coding. Next, quantitative analyses of visual characteristics (*channel*, *marks*, etc.) were performed stratified per *attribute*, thereby adding the visual design space to the vocabulary. Linking the vocabulary and design space enabled the creation of a visual dictionary to help identify *attributes* (e.g., resistance) with associated *channels* (e.g., points and lines on a common scale).

### Qualitative Analysis

Comments about the visualizations were coded in Microsoft Excel by two researchers (CL and JK). An open coding round was followed by axial coding to discover related concepts in the sub-codes. Differences were discussed until consensus was reached, which increased the internal validity (Patton, 1999). Next to improvements, CL and JK coded remarks about chart junk (i.e., the unnecessary and/or redundant use of visualization embellishments) (Tufte, 2001).

## Results

In total, 150 visualizations were analyzed (IRR: 87% joint probability of agreement). The following sections are separated into visual vocabulary (content) and dictionary with results stratified by identified *attributes*. These sections are followed by visualization ratings, identified visualization problems, and suggested recommendations for visualization creators.

### Visual Vocabulary (Content)

In total, 48 different attributes were coded. The 10 most used attributes were *time* (*n* = 69, 46.0%), *setting* (*n* = 43, 28.7%), *antimicrobial consumption* (*n* = 32, 21.3%), *resistance* (*n* = 31, 20.1%), *antimicrobials* (*n* = 27, 18.0%), *percentage* (*n* = 26, 17.3%), *count* (*n* = 24, 16.0%), *incidence* (*n* = 24, 16.0%), *numeric value* (*n* = 20, 13.3%), and *bacteria* (*n* = 12, 8.0%). Attributes could be grouped into objects (e.g., *bacteria*) and measurements (e.g., *percentage*). However, the following analysis focuses on attribute combinations and attributes are thus kept ungrouped.

**FIGURE 3:**
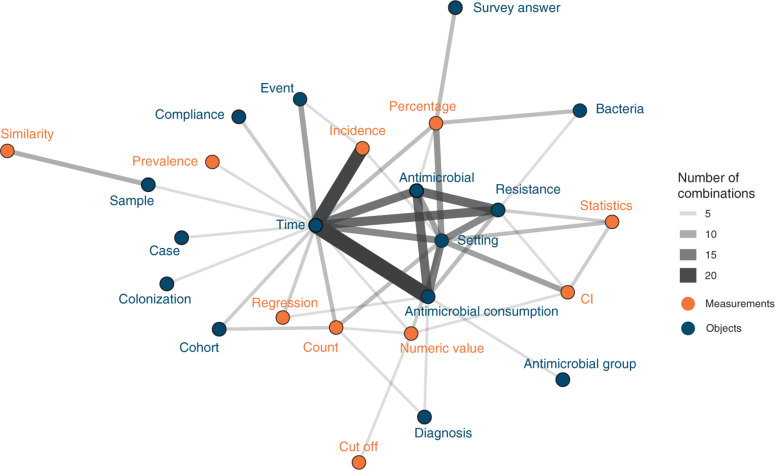
Attribute combinations in visualizations (combination count ≥3), thickness of lines corresponds to combination count. Orange points and labels represent attributes related to measurements; blue points and labels represent attributes related to objects.

Attributes showed different co-occurrence patterns (Figure 3). The 10 most frequent combinations were *time* and *antimicrobial consumption* (*n* = 21), *time* and *incidence* (*n* = 18), *antimicrobial consumption* and *antimicrobials* (*n* = 12), *antimicrobials* and *resistance* (*n* = 12), *time* and *resistance* (*n* = 12), *time* and *antimicrobials* (*n* = 11), *antimicrobial consumption* and *setting* (*n* = 10), *resistance* and *setting* (*n* = 9), *time* and *setting* (*n* = 9), and *percentage* and *setting* (*n* = 8).

### Visual Dictionary

#### Visualization Types

Fourteen different visualization types were identified of which bar charts (*n* = 54, 36.0%) and line charts (*n* = 42, 28.1%) were predominantly used. Bar charts were most frequently associated with attributes *antimicrobials*, *bacteria*, *cohorts*, *compliance*, *counts*, *diagnosis*, *errors*, *percentages*, *resistance*, *setting*, and *survey answers*. Line charts were predominantly associated with *antimicrobial consumption*, *costs*, *cut-off*, *incidence*, *numeric values*, *regression*, *statistics*, and *time* (detailed results available in Supplementary Material S3).

Different visualization types combined in one visualization were used in 10.7% (*n* = 16) of all visualizations. In these, visualization types that were combined more than once were bar charts with line charts (*n* = 5, 31.3%) and stacked bar charts with line charts (*n* = 2, 12.5%). In 41 visualizations (27.3%) facets were used, i.e., one visualization split into a matrix of visualizations using the same axes.

#### Visual Design Space

Different patterns of visual characteristics could be identified for different *attributes* (detailed counts and percentages in Supplementary Material S4).

1. Position: Horizontal axes were mostly used for *Antimicrobials*, *bacteria*, *confidence intervals*, *counts*, *cut-offs*, *diagnoses*, *events*, *numeric values*, *settings*, *similarity*, and *time*. In contrast, vertical axes were mostly used for *antimicrobial consumption*, *cases*, *cohorts*, *counts*, *errors*, *incidence*, *percentages*, *regression, resistance*, *samples*, *statistics*, and *survey answers.*
2. Marks, color, shape: *Attributes* also differed in their use of marks. Some attributes had clear associations with mark types, e.g., *time* was always visualized with lines. Area marks were seldomly used, e.g., for *antimicrobial consumption*, *counts*, *cut-offs*, *incidence*, *numeric values*, *percentages*, and *resistance*. Color and shape channels were frequently used in most attributes. A detailed color and shape channel analysis is available in the Supplementary Material S4.
3. Size: Size was most often visually reflected through length. Area to reflect size was used for *antimicrobial consumption*, *count*, *cut-off*, *incidence*, *numeric values*, *percentages*, and *resistance*. Volume was rarely used (for *count* and *percentages*).
4. Magnitude/ordering: Position on a common scale was mostly used in quantitative and ordered attributes reflecting the best channel effectiveness for these attribute types. Categorical attributes mostly used color hue, which is preferred over the less effective use of shapes. A detailed channel effectiveness analysis is available in the Supplementary Material S5.

### Ratings, Problems, and Chart Junk

#### Visualization Ratings

Overall, 55.3% (*n* = 83) of all visualizations were interpretable without additional text (in caption or in the manuscript text). The overall choice of visualization type was rated with a mean of 4.62 (SD: 0.9) on a scale from 1 (poor) to 5 (good). The assessment of the visualization quality (scale 1 = poor to 5 = good) was rated with a mean of 3.6 (SD:1.2).

#### Identified Problems

The coding of the identified problems are presented in the coding scheme in Table 1, including axial codes, open codes, and frequencies. In Supplementary Material S6, additional illustrative quotes per code are presented.

**TABLE 1:** Identified problems in stewardship data visualization.

**Code (axial)**	**Code (open)**	**Count**
Missing labels, annotations, legend and/or abbreviation explanations	Legend/caption is missing/unclear	26
	Labels for lines/points are missing/unclear	23
	Labels for axes are missing/unclear	20
	Annotation/direct labeling overflow	14
	Abbreviations not explained	12
	Use of colors not explained	7
Subtotal		102
Axes not readable	Axis intervals uneven (within visualization and between faceted visualizations)	17
	Axes texts not clearly readable	11
	Too short/dense axes/intervals	5
	Uneven bar placement	1
	Axis intervals illogical (within visualization and between faceted visualizations)	1
Subtotal		35
Color scheme mismatch	Groups not distinguishable by colors	14
	Non-intuitive color schemes used	6
	Categorical colors used for ordered attribute	5
	Groups not distinguishable from background	2
Subtotal		27
Hidden data points by overlaps	Overlap of shapes problematic	7
Subtotal		7
Using suboptimal channel effectiveness	Groups not distinguishable by shapes	12
	Sub-effective channel is chosen	3
Subtotal		15
Size scale indistinguishable	Differences in size not clear	10
	Groups not distinguishable by shape size	3
	Contrasts between groups not clear	2
Subtotal		15
Missing channel	Line types not used to distinguish between groups	2
	Colors not used to compare between visualization/groups	2
Subtotal		4
Visualization type does not (optimally) fit data	Other visualization type preferred	21
Subtotal		21
Data points/lines on double axes	Double *Y*-axes difficult to read	11
Subtotal		11
Channel overflow	Double use of shape and color	8
	Unnecessary use of shape sizes	1
	Unnecessary use of color	1
	Too many colors	1
Subtotal		11
Attribute overflow	Too many attributes	2
	Relating attributes that are not related	1
Subtotal		3
Information sparsity	Could be text	1
Subtotal		1
Incoherent ordering	Data not ordered coherently	1
Subtotal		1
Grand total		253

#### Chart Junk

Most chart junk represented text that cluttered the visualization (*n* = 8), for example with redundant direct labels for each data point. Other chart junk was found in visualizations using unnecessary 3D (*n* = 8), background colors (*n* = 6), shadow (*n* = 4), and color/shape filling (*n* = 4).

### Examples and Recommendations

**FIGURE 4:**
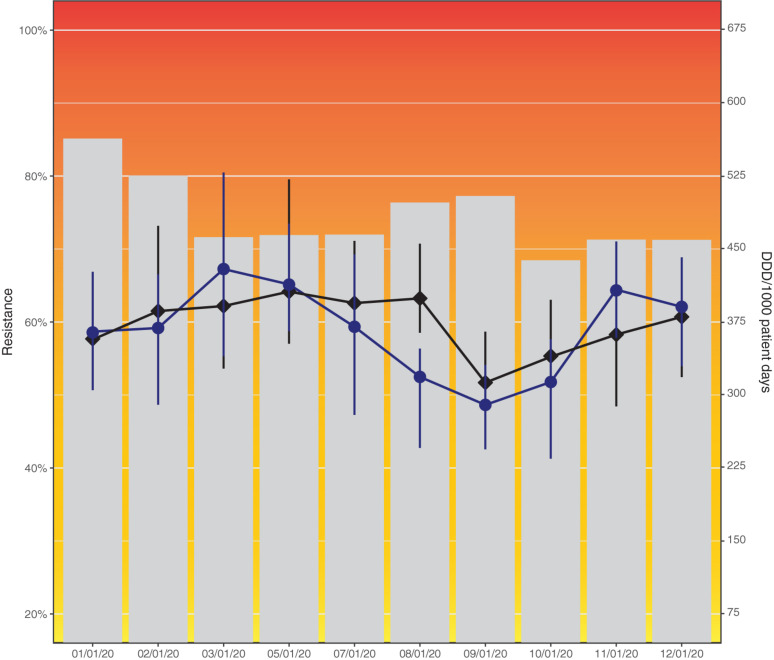
Resistance to amoxicillin in *Escherichia coli* and consumption of cefuroxime (black) and piperacillin/tazobactam (blue) across hospital departments in 2020. This data visualization (simulated data) shows several problems identified in this study: Axes not starting at zero, use of double *y*-axes, background colors, hidden data points by overlaps, color scheme mismatch (blue and black difficult to distinguish), unequal axis steps on *x*-axis, missing legend, incomplete axis labels (abbreviation not explained).

**FIGURE 5:**
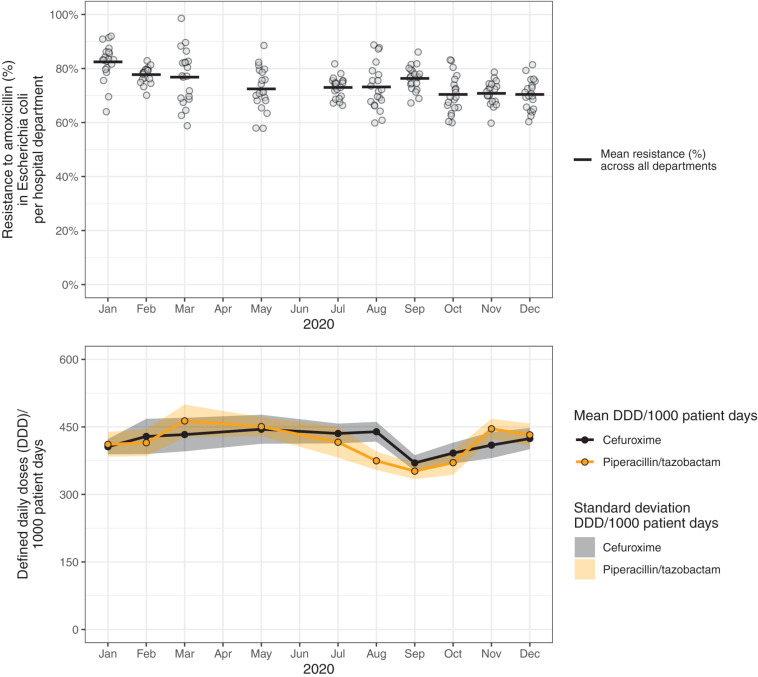
Resistance to amoxicillin in *Escherichia coli* and consumption of cefuroxime and piperacillin/tazobactam across hospital departments in 2020. These data visualizations use the same data as in Figure 4 (simulated data), but propose an improved visualization.

To illustrate problems in data visualization, we designed a visualization that exhibits several of the identified problems based on simulated data (Figure 4). Figure 5 proposes an alternative to Figure 4 where the identified problems were avoided. Of note, data such as the simulated data in these figures can be visualized in many different ways, depending on the underlying research questions.

**FIGURE 6:**
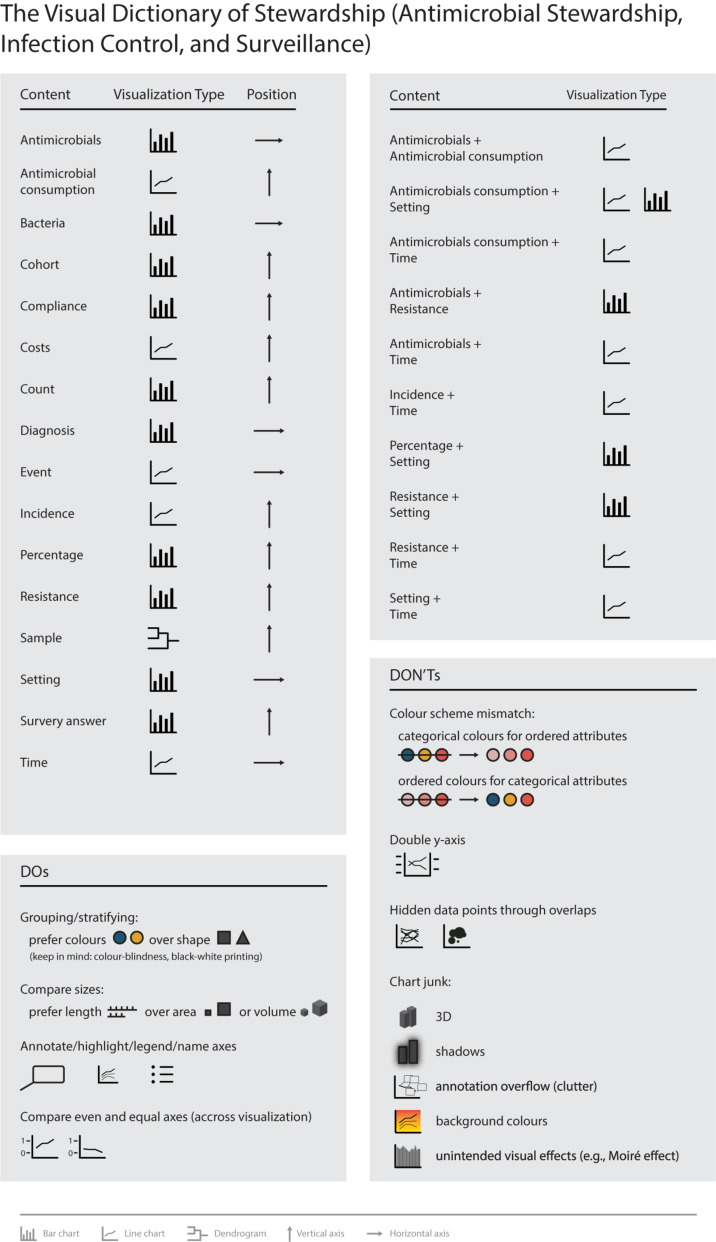
The visual dictionary of stewardship (antimicrobial stewardship, infection control, and institutional surveillance).

Figure 6 summarizes the results of this study and presents the visual dictionary of stewardship. In addition, it provides a set of recommendations to avoid the most common problems in data visualizations as identified in this study.

## Discussion

This study systematically analyzed the visual domain context of stewardship, i.e., antimicrobial stewardship, infection control, and institutional surveillance. Stewardship experts and scientists that create data visualizations can benefit from the revealed visual domain context, since it allows them to anticipate the visual habits of their target audience. The results of this study can serve as the basis to inform visualization creators to optimize visual communication in the field and to guide user-centered design, e.g., in clinical decision support systems.

### Findings and Future Directions

With the systematic analysis of the visual domain context of stewardship we revealed common practices and identified problems with current implemented visualizations. In general, the use of data visualizations for communicating data is highly encouraged. It greatly supports the interpretation, memorization, and communication of insights and knowledge gained from data. In this study, we identified 14 different visualization types used in the visual domain context of the field. However, more than 80% of all visualizations used classical (stacked) bar or line charts; quite homogenous design choices. We argue that the visualization type choice is based on tradition and habits as a systematic approach to data visualization in the field was missing until now (Salinas et al., 2020). A lack of awareness and knowledge about data visualization design and design alternatives might lead to suboptimal data visualizations. Examples from our findings were the use of less effective visual channels, suboptimal plot types for the presented data, or mismatches in color choices for different data types. Similar visualization pitfalls were identified in studies focusing on common visualization pitfalls in multidisciplinary research related to visual representations and for environmental data, emphasizing instances where data visualization creators require more support in visualization design choices (Kelleher and Wagener, 2011; Bresciani and Eppler, 2015). Now that we revealed common data visualization practices in the visual dictionary for stewardship by linking often used attributes (i.e., content) and associated design choices (e.g., visualization type or marks), visualization creators in the field can match their visualizations with the audience’s visual expectations and habits.

However, given the wide variety of data in the field and the increased complexity that big data will add (in terms of volume, velocity, variety, veracity, validity, volatility, and value), more “visual variability” might be expected and even needed in the future (Khan et al., 2014; Gotz and Borland, 2016; Galletta et al., 2019). A first step toward visual variability is informing and teaching visualization creators and users about data visualization design alternatives. We see a clear role here for data visualization experts and software developers to cocreate open-source/access tools that support visualization creators in their visualization choices (e.g., reminders for adding labels and legends, suggestions for optimal color schemes, warnings in case of chart junk). Our results and findings from similar studies in other fields can support them in doing so by providing an overview of common data visualization practices in the field, including dos and don’ts (Crisan et al., 2019; Nusrat et al., 2019).

Of note, academic journals play an important part in this process by providing the platform for data visualizations and should be encouraged to promote high quality data visualization practices. Furthermore, it could be worth considering standardizing data visualization for often used data types and contents in the field, given the prominent patterns in the visual dictionary (e.g., time series were part of 43.3% of all studied visualizations) within the large variety of content (48 different attributes such as antimicrobials, bacteria, or time) observed in this study. For time series specifically, an overview of data visualization methods exist (Fang et al., 2020), and similar standardizing initiatives can be found in the AMR field [e.g., European Committee of Antimicrobial Susceptibility Testing (EUCAST; Brown et al., 2015)] and other fields [e.g., the Intergovernmental Panel on Climate Change (IPCC) and standardized medical data visualization based on the ISO13606 data model (Kopanitsa et al., 2015; Gomis and Pidcock, 2018)]. This could help ensure high quality data visualizations for reliable insights in AMR/stewardship related data.

In the light of growing complexities and increasing data volumes, genomic data and their visualization play a special role in the field. Although genomic data visualizations were included in this study, most visualizations were simple dendograms and phylogenetic trees. As with the *a priori* excluded geographical data, these complex data require dedicated research and visualization techniques which are provided in great detail by others (see e.g., Crisan et al., 2019; Nusrat et al., 2019). An additional important aspect for high quality data visualization in the stewardship and AMR data field is the visualization of uncertainties. The visualization of uncertainties was not within the scope of this study and further research into the optimal display of uncertainty is highly encourage. For more information readers are referred to the work of others (Wilke, 2019; Korporaal et al., 2020; Padilla et al., 2020).

Studying the visual domain context is as important as studying data visualizations themselves. The importance of assessing visual habits and perceptions in data visualization has been demonstrated before in other medical fields revealing that personal preferences and visualization habits might not always match with novel data visualization approaches and recommendations (Backonja et al., 2018; Aung et al., 2019b). Aung et al. (2019b) published an exemplary study in the field of reproductive, maternal, new-born and child health, focusing on data visualization interpretation capacity and preferences in their target audience by combining interviews on interpretability and card-sorting of preferred visualizations. Thus, for data visualization in general we strongly believe that incorporating best practices is essential, but advocate that these should be carefully balanced with visual habits and expectations in the field and the message to be conveyed. Additionally, research is needed to better understand how data visualizations in general impact the viewers in terms of changes in opinions or attitudes that direct decision-making or behavior changes (Pandey et al., 2014).

In future research special attention should be paid to matching the visual dictionary and the context in which the visualization will be used in terms of users, their tasks and current practices (e.g., studying questions like “How do current visualizations support to do current tasks?” and “What visualizations would the target audience like to see?”) (Lam et al., 2012). This also includes color-blindness considerations, as extensively studied by others (Ahmed et al., 2020; Crameri et al., 2020; Burggraaff et al., 2021). We see a clear parallel with user-centered eHealth design that emphasizes the need for a holistic understanding of the interrelations between technology, people, and their context (van Gemert-Pijnen et al., 2018). Both qualitative (e.g., interviews) and quantitative (e.g., eye-tracking in current data visualizations) study designs can contribute to such a holistic understanding, which in turn can inform or improve the design of visualizations (or eHealth) in terms of required content, functionalities, and usability (Keizer et al., 2020). Therefore, complementing research on data visualizations, as the current study and many other studies do, with research that primarily focuses on the interaction between people, their context and how data visualizations can support them, is needed (Lam et al., 2012).

### Limitations and Strengths

This study has several limitations. Despite sampling from a comprehensive set of articles that cover the stewardship field, only a limited number of data visualizations were included. Moreover, only data visualizations from scientific publications and not from other sources relevant to stewardship data visualization creators [e.g., data systems used in practice (Huber et al., 2018; Sedrakyan et al., 2019) and AMR policy reports (Anderson et al., 2019; World Health Organization, 2020a)] were included. Therefore, we missed data visualizations for other stewardship content, making our findings potentially more applicable to stewardship researchers than healthcare professionals. However, the observed homogeneity of data visualization types suggests saturation regarding the visual design space for stewardship. Subsequent research into the visual domain context of stewardship should include these additional sources to ensure a more comprehensive picture for healthcare professionals. Even though the extracted data visualizations were systematically analyzed using a structured assessment form based on existing data visualization nomenclature and categorization (Munzner, 2014), the analyses relied on the subjective interpretation and rating by the coding researchers. Several measures were taken to validate our findings, including discussing the assessment form and results within a multidisciplinary team of data-visualization and AMR experts, analyzing the interrater-reliability, and comparing our findings to other data visualization studies. Our study is one of the first empirical studies that explores the use of data visualization in stewardship, thereby adding to the few review studies providing primers for data visualization recommendations and best practices in the stewardship field (Carroll et al., 2014; Salinas et al., 2020). Furthermore, our structured assessment approach can be applied in future studies in the broader One Health field to unravel the visual dictionary of the fields of human, animal, and ecosystem health, considering interdisciplinary differences in data and data visualizations and their integration and interpretation (Lapinski et al., 2015; European Centre for Disease Prevention and Control [ECDC], European Food Safety Authority [EFSA], and European Medicines Agency [EMA], 2017; EFSA, 2021).

## Conclusion

In this study, we analyzed the visual domain context of stewardship (antimicrobial stewardship, infection control, and institutional surveillance). We successfully created a visual dictionary that can support the process of creating and using tailor-made data visualizations in the field. Thereby, our results allow data visualization creators to learn the *visual language* of the diverse field of stewardship. As data-driven solutions for stewardship are of increasing importance, effective processes of transforming this data to insights and knowledge is essential. Data visualization supports and enables this transformation and our results can guide the optimal visualization design choices that are grounded on expectations and habits in the field. In the future, our study can provide the basis to further expand the visual dictionary of antimicrobial stewardship toward more effective data visualizations that improve data insights, knowledge, and decision-making.

## Data Availability Statement

The original contributions presented in the study are included in the article/Supplementary Material, further inquiries can be directed to the corresponding author/s.

## Author Contributions

JK and CL: conceptualization, data analysis, and writing – original draft. CL: visualization. JK, CL, NB-dJ, and CG: discussing assessment form and results. NB-dJ, BS, LG-P, CA, and CG: supervision. All authors: writing – review and editing.

## Conflict of Interest

The authors declare that the research was conducted in the absence of any commercial or financial relationships that could be construed as a potential conflict of interest.

## Publisher’s Note

All claims expressed in this article are solely those of the authors and do not necessarily represent those of their affiliated organizations, or those of the publisher, the editors and the reviewers. Any product that may be evaluated in this article, or claim that may be made by its manufacturer, is not guaranteed or endorsed by the publisher.
