# Supplementary material for: The Visual Dictionary of Antimicrobial Stewardship, Infection Control, and Institutional Surveillance Data
Source: Front Microbiol. 2021 Oct 28;12:743939. doi: 10.3389/fmicb.2021.743939 (PMC8581675; doi:10.3389/fmicb.2021.743939)
Supplement: Supplementary file 1 [file Data_Sheet_1.docx]

Supplementary Material

# References for the assessed 150 articles

Stewardship: [1-50]

1. Abbo, L., et al., *Faculty and Resident Physicians’ Attitudes, Perceptions, and Knowledge about Antimicrobial Use and Resistance.* Infection Control and Hospital Epidemiology, 2011. **32**(7): p. 714-18.

2. Alexa Oniciuc, E.A., et al., *A European questionnaire-based study on population awareness and risk perception of antimicrobial resistance.* FEMS Microbiol Lett, 2019. **366**(17).

3. Alumran, A., X. Hou, and C. Hurst, *Assessing the overuse of antibiotics in children in Saudi Arabia: validation of the parental perception on antibiotics scale (PAPA scale).* Health and Quality of Life Outcomes, 2013. **11**(39).

4. Assimacopoulos, A., et al., *A brief retrospective review of medical records comparing outcomes for inpatients treated via telehealth versus in-person protocols: is telehealth equally effective as in-person visits for treating neutropenic fever, bacterial pneumonia, and infected bacterial wounds?* Telemed J E Health, 2008. **14**(8): p. 762-8.

5. Beatty, M.E., et al., *Sporadic paediatric diarrhoeal illness in urban and rural sites in Nyanza Province, Kenya.* East African Medical Journal, 2009. **86**(8): p. 387-98.

6. Bhalla, N., et al., *Introducing an antibiotic stewardship program in a humanitarian surgical hospital.* Am J Infect Control, 2016. **44**(11): p. 1381-1384.

7. Budd, E., et al., *Adaptation of the WHO Essential Medicines List for national antibiotic stewardship policy in England: being AWaRe.* J Antimicrob Chemother, 2019. **74**(11): p. 3384-3389.

8. Chan, Y.H., et al., *Antibiotics nonadherence and knowledge in a community with the world's leading prevalence of antibiotics resistance: implications for public health intervention.* Am J Infect Control, 2012. **40**(2): p. 113-7.

9. Chopra, R., et al., *An audit of antimicrobial prescribing in an acute dental care department.* Primary Dental Journal, 2014. **3**(4): p. 24-9.

10. Cooke, J., et al., *Narrative review of primary care point-of-care testing (POCT) and antibacterial use in respiratory tract infection (RTI).* BMJ Open Respir Res, 2015. **2**(1): p. e000086.

11. Coupat, C., et al., *Selective reporting of antibiotic susceptibility data improves the appropriateness of intended antibiotic prescriptions in urinary tract infections: a case-vignette randomised study.* Eur J Clin Microbiol Infect Dis, 2013. **32**(5): p. 627-36.

12. Doernberg, S.B., V. Dudas, and K.K. Trivedi, *Implementation of an antimicrobial stewardship program targeting residents with urinary tract infections in three community long-term care facilities: a quasi-experimental study using time-series analysis.* Antimicrob Resist Infect Control, 2015. **4**: p. 54.

13. Dyar, O.J., et al., *Do medical students feel prepared to prescribe antibiotics responsibly? Results from a cross-sectional survey in 29 European countries.* J Antimicrob Chemother, 2018. **73**(8): p. 2236-2242.

14. Ferson, K., et al., *Reliability of self-reporting of antibiotic consumption in the community - Index of Reliability.* J Clin Pharm Ther, 2014. **39**(5): p. 468-70.

15. Fuchs, A., et al., *Reviewing the WHO guidelines for antibiotic use for sepsis in neonates and children.* Paediatr Int Child Health, 2018. **38**(sup1): p. S3-S15.

16. Garcia-Rodriguez, J.F., et al., *Meropenem antimicrobial stewardship program: clinical, economic, and antibiotic resistance impact.* Eur J Clin Microbiol Infect Dis, 2019. **38**(1): p. 161-170.

17. Gualano, M.R., et al., *General population's knowledge and attitudes about antibiotics: a systematic review and meta-analysis.* Pharmacoepidemiol Drug Saf, 2015. **24**(1): p. 2-10.

18. Howe, P. and J. Adams, *Reducing urinary tract infections in catheterised patients.* Nursing Standard, 2015. **29**(20): p. 43-9.

19. Khare, S., et al., *Antibiotic Prescribing by Informal Healthcare Providers for Common Illnesses: A Repeated Cross-Sectional Study in Rural India.* Antibiotics (Basel), 2019. **8**(3).

20. Labi, A.K., et al., *Physicians' knowledge, attitudes, and perceptions concerning antibiotic resistance: a survey in a Ghanaian tertiary care hospital.* BMC Health Serv Res, 2018. **18**(1): p. 126.

21. Lambert, M.F., G.A. Masters, and S.L. Brent, *Can mass media campaigns change antimicrobial prescribing? A regional evaluation study.* J Antimicrob Chemother, 2007. **59**(3): p. 537-43.

22. Lechtenberg, R.J., et al., *Variation in adherence to the treatment guidelines for Neisseria gonorrhoeae by clinical practice setting, California, 2009 to 2011.* Sex Transm Dis, 2014. **41**(5): p. 338-44.

23. Lee, C.R., et al., *Strategies to minimize antibiotic resistance.* Int J Environ Res Public Health, 2013. **10**(9): p. 4274-305.

24. Ljung, R., et al., *Inequality in quality? Regional and educational differences in treatment with fluoroquinolone in urinary tract infection of 236,376 Swedish patients.* BMJ Qual Saf, 2011. **20**(1): p. 9-14.

25. Malo-Fumanal, S., et al., *Differences in outpatient antibiotic use between a Spanish region and a Nordic country.* Enferm Infecc Microbiol Clin, 2014. **32**(7): p. 412-7.

26. McKinnon, P.S., A.J. Boening, and A.N. Amin, *Optimizing delivery of care for patients with MRSA infection: focus on transitions of care.* Hosp Pract (1995), 2011. **39**(2): p. 18-31.

27. Mills, H.L., et al., *Evaluation of metrics for benchmarking antimicrobial use in the UK dairy industry.* Vet Rec, 2018. **182**(13): p. 379.

28. Parker, H.M. and K. Mattick, *The determinants of antimicrobial prescribing among hospital doctors in England: a framework to inform tailored stewardship interventions.* Br J Clin Pharmacol, 2016. **82**(2): p. 431-40.

29. Pulcini, C., et al., *Junior doctors’ knowledge and perceptions of antibiotic resistance and prescribing: a survey in France and Scotland.* Clinical Microbiology and Infectious Diseases, 2010. **17**: p. 80-7.

30. Rello, J., et al., *International Conference for the Development of Consensus on the Diagnosis and Treatment of Ventilator-associated Pneumonia.* Chest, 2001. **120**(3): p. 955-70.

31. Rennert-May, E., et al., *Clinical practice guidelines for creating an acute care hospital-based antimicrobial stewardship program: A systematic review.* Am J Infect Control, 2019. **47**(8): p. 979-993.

32. Rodriguez Sanchez, F., et al., *Antibiotic prophylaxis habits in oral implant surgery among dentists in Italy: a cross-sectional survey.* BMC Oral Health, 2019. **19**(1): p. 265.

33. Schulze-Geisthovel, S.V., et al., *Survey on the risk awareness of german pig and cattle farmers in relation to dealing with MRSA and antibiotics.* Infect Ecol Epidemiol, 2016. **6**: p. 29817.

34. Sheng, T., et al., *Point-prevalence study of antimicrobial use in public hospitals in southern Sri Lanka identifies opportunities for improving prescribing practices.* Infect Control Hosp Epidemiol, 2019. **40**(2): p. 224-227.

35. Simoes, A.S., et al., *Participatory implementation of an antibiotic stewardship programme supported by an innovative surveillance and clinical decision-support system.* J Hosp Infect, 2018. **100**(3): p. 257-264.

36. Spoorenberg, V., et al., *Adequacy of an evidence-based treatment guideline for complicated urinary tract infections in the Netherlands and the effectiveness of guideline adherence.* Eur J Clin Microbiol Infect Dis, 2013. **32**(12): p. 1545-56.

37. Stille, C.J., et al., *Increased use of second-generation macrolide antibiotics for children in nine health plans in the United States.* Pediatrics, 2004. **114**(5): p. 1206-11.

38. Stratchounski, L.S., et al., *The Inventory of Antibiotics in Russian Home Medicine Cabinets.* Clinical Infectious Diseases, 2003. **37**: p. 498-505.

39. Summers, J.F., A. Hendricks, and B. D.C., *Prescribing practices of primary-care veterinary practitioners in dogs diagnosed with bacterial pyoderma.* BMC Veterinary Research, 2014. **10**(240).

40. Tangden, T., et al., *Radical reduction of cephalosporin use at a tertiary hospital after educational antibiotic intervention during an outbreak of extended-spectrum beta-lactamase-producing Klebsiella pneumoniae.* J Antimicrob Chemother, 2011. **66**(5): p. 1161-7.

41. Thillaivanam, S., et al., *The effectiveness of the McIsaac clinical decision rule in the management of sore throat: an evaluation from a pediatrics ward.* Pediatr Res, 2016. **80**(4): p. 516-20.

42. Uda, K., et al., *Targets for Optimizing Oral Antibiotic Prescriptions for Pediatric Outpatients in Japan.* Jpn J Infect Dis, 2019. **72**(3): p. 149-159.

43. Varonen, H., et al., *Implementing guidelines on acute maxillary sinusitis in general practice--a randomized controlled trial.* Fam Pract, 2007. **24**(2): p. 201-6.

44. Wagstaff, B., *Impact of antibiotic restrictions: the patient’s perspective.* Clinical Microbiology and Infectious Diseases, 2006. **12**: p. 10-5.

45. Wang, S., et al., *The impact of the national action plan on the epidemiology of antibiotic resistance among 352,238 isolates in a teaching hospital in China from 2015 to 2018.* Antimicrob Resist Infect Control, 2019. **8**: p. 22.

46. Weiss, K., et al., *Impact of a multipronged education strategy on antibiotic prescribing in Quebec, Canada.* Clin Infect Dis, 2011. **53**(5): p. 433-9.

47. Willemsen, I., et al., *Appropriateness of antimicrobial therapy measured by repeated prevalence surveys.* Antimicrob Agents Chemother, 2007. **51**(3): p. 864-7.

48. Young, I., et al., *Knowledge and Attitudes toward Food Safety and Use of Good Production Practices among Canadian Broiler Chicken Producers.* Journal of Food Protection, 2010. **73**(7): p. 1278-87.

49. Zoorob, R., et al., *Nonprescription Antimicrobial Use in a Primary Care Population in the United States.* Antimicrob Agents Chemother, 2016. **60**(9): p. 5527-32.

50. Zoutman, D.E., et al., *The state of infection surveillance and control in Canadian acute care hospitals.* Am J Infect Control, 2003. **31**(5): p. 266-72.

Institutional surveillance: [51-100]

51. Agarwal, M. and E.L. Larson, *Risk of drug resistance in repeat gram-negative infections among patients with multiple hospitalizations.* J Crit Care, 2018. **43**: p. 260-264.

52. Aizawa, Y., et al., *Antimicrobial Stewardship Program in a Pediatric Intensive Care Unit.* J Pediatric Infect Dis Soc, 2018. **7**(3): p. e156-e159.

53. Aldeyab, M.A., et al., *Modelling the impact of antibiotic use and infection control practices on the incidence of hospital-acquired methicillin-resistant Staphylococcus aureus: a time-series analysis.* J Antimicrob Chemother, 2008. **62**(3): p. 593-600.

54. Bai, B., et al., *Linezolid Consumption Facilitates the Development of Linezolid Resistance in Enterococcus faecalis in a Tertiary-Care Hospital: A 5-Year Surveillance Study.* Microb Drug Resist, 2019. **25**(6): p. 791-798.

55. Bauer, K.A., et al., *Extended-infusion cefepime reduces mortality in patients with Pseudomonas aeruginosa infections.* Antimicrob Agents Chemother, 2013. **57**(7): p. 2907-12.

56. Bilir, S.P., et al., *The economic impact of rapid Candida species identification by T2Candida among high-risk patients.* Future Microbiol, 2015. **10**(7): p. 1133-44.

57. Boyles, T.H., et al., *Sustained reduction in antibiotic consumption in a South African public sector hospital; Four year outcomes from the Groote Schuur Hospital antibiotic stewardship program.* S Afr Med J, 2017. **107**(2): p. 115-118.

58. Bruinsma, N., et al., *Influence of population density on antibiotic resistance.* J Antimicrob Chemother, 2003. **51**(2): p. 385-90.

59. Camus, C., et al., *Decline of multidrug-resistant Gram negative infections with the routine use of a multiple decontamination regimen in ICU.* J Infect, 2016. **73**(3): p. 200-9.

60. Chong, Y., et al., *Korean Nationwide Surveillance of Antimicrobial Resistance of bacteria in 1997.* Yonsei Med J, 1998. **39**(6): p. 569-77.

61. Cummings, K.J., et al., *Antimicrobial resistance trends among Salmonella isolates obtained from horses in the northeastern United States (2001–2013).* Am J Vet Res, 2015. **77**(5): p. 505-13.

62. de With, K., et al., *Antibiotic use in German university hospitals 1998-2000 (Project INTERUNI-II).* Int J Antimicrob Agents, 2004. **24**(3): p. 213-8.

63. Eagye, K.J. and D.P. Nicolau, *Change in antipseudomonal carbapenem susceptibility in 25 hospitals across 9 years is not associated with the use of ertapenem.* J Antimicrob Chemother, 2011. **66**(6): p. 1392-5.

64. El-Nawawy, A., et al., *Incidence of Multidrug-Resistant Organism Among Children Admitted to Pediatric Intensive Care Unit in a Developing Country.* Microb Drug Resist, 2018. **24**(8): p. 1198-1206.

65. Giacobbe, D.R., et al., *Reduced Incidence of Carbapenem-Resistant Klebsiella pneumoniae Infections in Cardiac Surgery Patients after Implementation of an Antimicrobial Stewardship Project.* Antibiotics (Basel), 2019. **8**(3).

66. Goryachkina, K., et al., *Quality use of medicines: a new method of combining antibiotic consumption and sensitivity data--application in a Russian hospital.* Pharmacoepidemiol Drug Saf, 2008. **17**(6): p. 636-44.

67. Grau, S., et al., *How to measure and monitor antimicrobial consumption and resistance.* Enfermedades Infecciosas y Microbiología Clínica, 2013. **31**: p. 16-24.

68. Gurcuoglu, E., et al., *Epidemiology of nosocomial candidaemia in a university hospital: a 12-year study.* Epidemiol Infect, 2010. **138**(9): p. 1328-35.

69. Japoni, A., et al., *Multidrug-Resistant Bacteria Isolated from Intensive-Care-Unit Patient Samples.* Braz J Infect Dis, 2009. **13**(2): p. 118-22.

70. Jensen, U.S., et al., *Consequences of increased antibacterial consumption and change in pattern of antibacterial use in Danish hospitals.* J Antimicrob Chemother, 2009. **63**(4): p. 812-5.

71. Jones, B.E., et al., *Trends in Antibiotic Use and Nosocomial Pathogens in Hospitalized Veterans with Pneumonia at 128 Medical Centers, 2006-2010.* Clin Infect Dis, 2015. **61**(9): p. 1403-10.

72. Lamoth, F., et al., *Comparison of hospital-wide and unit-specific cumulative antibiograms in hospital- and community-acquired infection.* Infection, 2010. **38**(4): p. 249-53.

73. Leibowitz, L.D., *MRSA burden and interventions.* Int J Antimicrob Agents, 2009. **34**(Suppl 3): p. S11-3.

74. Lepelletier, D. and H. Richet, *Surveillance and Control of Methicillin Resistant Staphylococcus aureus Infections in French Hospitals.* Infect Control Hosp Epidemiol, 2001. **22**(11): p. 677-82.

75. Liu, K.S., et al., *Antimicrobial resistance of bacterial isolates from respiratory care wards in Taiwan: a horizontal surveillance study comparison of the characteristics of nosocomial infection and antimicrobial-resistant bacteria in adult Intensive Care Units and two respiratory care facilities for mechanically ventilated patients at a tertiary care centre in Taiwan.* Int J Antimicrob Agents, 2011. **37**(1): p. 10-5.

76. Lob, S.H., et al., *Epidemiology and Antimicrobial Susceptibility of Gram-Negative Pathogens Causing Intra-abdominal Infections in Pediatric Patients in Europe-SMART 2011-2014.* J Pediatric Infect Dis Soc, 2017. **6**(1): p. 72-79.

77. Ma, X., et al., *Antimicrobial stewardship of Chinese ministry of health reduces multidrug-resistant organism isolates in critically ill patients: a pre-post study from a single center.* BMC Infect Dis, 2016. **16**(1): p. 704.

78. Meyer, E., et al., *Is the prevalence of Stenotrophomonas maltophilia isolation and nosocomial infection increasing in intensive care units?* Eur J Clin Microbiol Infect Dis, 2006. **25**(11): p. 711-4.

79. Montagnani, F., et al., *Erythromycin resistance in Streptococcus pyogenes and macrolide consumption in a central Italian region.* Infection, 2009. **37**(4): p. 353-7.

80. Murillo, O., et al., *The changing epidemiology of bacteraemic osteoarticular infections in the early 21st century.* Clin Microbiol Infect, 2015. **21**(3): p. 254 e1-8.

81. Opatowski, M., et al., *Hospitalisations with infections related to antimicrobial-resistant bacteria from the French nationwide hospital discharge database, 2016.* Epidemiol Infect, 2019. **147**: p. e144.

82. Pena, C., et al., *Nosocomial outbreak of a non-cefepime-susceptible ceftazidime-susceptible Pseudomonas aeruginosa strain overexpressing MexXY-OprM and producing an integron-borne PSE-1 betta-lactamase.* J Clin Microbiol, 2009. **47**(8): p. 2381-7.

83. Peterson, L.R., et al., *Nonimpact of Decolonization as an Adjunctive Measure to Contact Precautions for the Control of Methicillin-Resistant Staphylococcus aureus Transmission in Acute Care.* Antimicrob Agents Chemother, 2016. **60**(1): p. 99-104.

84. Piednoir, E., et al., *Long-term clinical and economic benefits associated with the management of a nosocomial outbreak resulting from extended-spectrum beta-lactamase-producing Klebsiella pneumoniae.* Crit Care Med, 2011. **39**(12): p. 2672-7.

85. Randhawa, V., et al., *Weighted-incidence syndromic combination antibiograms to guide empiric treatment of critical care infections: a retrospective cohort study.* Crit Care, 2014. **18**(3): p. R112.

86. Reinert, R.R., et al., *Emergence of macrolide and penicillin resistance among invasive pneumococcal isolates in Germany.* J Antimicrob Chemother., 2002. **49**(1): p. 61-8.

87. Ryan, R.J., C. Lindsell, and P. Sheehan, *Fluoroquinolone resistance during 2000-2005: an observational study.* BMC Infect Dis, 2008. **8**: p. 71.

88. Santana Rde, C., et al., *Secular trends in Klebsiella pneumoniae isolated in a tertiary-care hospital: increasing prevalence and accelerated decline in antimicrobial susceptibility.* Rev Soc Bras Med Trop, 2016. **49**(2): p. 177-82.

89. Scholte, J.B., et al., *Empirical antibiotic therapy for pneumonia in intensive care units: a multicentre, retrospective analysis of potentially pathogenic microorganisms identified by endotracheal aspirates cultures.* Eur J Clin Microbiol Infect Dis, 2015. **34**(11): p. 2295-305.

90. Singh, S., et al., *A three-point time series study of antibiotic usage on an intensive care unit, following an antibiotic stewardship programme, after an outbreak of multi-resistant Acinetobacter baumannii.* Eur J Clin Microbiol Infect Dis, 2015. **34**(9): p. 1893-900.

91. Sipahi, O.R., et al., *Antibacterial resistance patterns and incidence of hospital-acquired Staphylococcus aureus bacteremia in a tertiary care educational hospital in Turkey: a perspective from 2001 to 2013.* Turk J Med Sci, 2017. **47**(4): p. 1210-1215.

92. Song, I., et al., *Increased use of third-generation cephalosporin antibiotics in the outpatient setting in Korean children and adolescents.* Pediatr Int, 2018. **60**(9): p. 803-810.

93. Sousa-Pinto, B., et al., *Clinical and economic burden of hospitalizations with registration of penicillin allergy.* Ann Allergy Asthma Immunol, 2018. **120**(2): p. 190-194 e2.

94. Sun, H.K., D.P. Nicolau, and J.L. Kuti, *Resource utilization of adults admitted to a large urban hospital with community-acquired pneumonia caused by Streptococcus pneumoniae.* Chest, 2006. **130**(3): p. 807-14.

95. Takesue, Y., et al., *Effect of antibiotic heterogeneity on the development of infections with antibiotic-resistant gram-negative organisms in a non-intensive care unit surgical ward.* World J Surg, 2006. **30**(7): p. 1269-76.

96. Thorpe, K.E., P. Joski, and K.J. Johnston, *Antibiotic-Resistant Infection Treatment Costs Have Doubled Since 2002, Now Exceeding $2 Billion Annually.* Health Aff (Millwood), 2018. **37**(4): p. 662-669.

97. Tissera, K., et al., *Spread of resistant gram negatives in a Sri Lankan intensive care unit.* BMC Infect Dis, 2017. **17**(1): p. 490.

98. Vatopoulos, A.C., V. Kalapothaki, and N.J. Legakis, *Bacterial Resistance to Ciprofloxacin in Greece: Results from the National Electronic Surveillance System.* Emerg Infect Dis. , 1999. **5**(3): p. 471-6.

99. Velickovic-Radovanovic, R., et al., *Analysis of antibiotic utilization and bacterial resistance changes in a surgical clinic of Clinical Centre, Nis.* J Clin Pharm Ther, 2012. **37**(1): p. 32-6.

100. Wang, S., et al., *Changes in antimicrobial susceptibility of commonly clinically significant isolates before and after the interventions on surgical prophylactic antibiotics (SPAs) in Shanghai.* Braz J Microbiol, 2018. **49**(3): p. 552-558.

101. Balslev, U., et al., *An Outbreak of Borderline Oxacillin-Resistant Staphylococcus aureus (BORSA) in a Dermatological Unit.* Microb Drug Resist, 2005. **11**(1): p. 78-81.

Infection control: [101-150]

102. Ben-David, D., et al., *Carbapenem-resistant Klebsiella pneumoniae in post-acute-care facilities in Israel.* Infect Control Hosp Epidemiol, 2011. **32**(9): p. 845-53.

103. Boldin, B., M.J. Bonten, and O. Diekmann, *Relative effects of barrier precautions and topical antibiotics on nosocomial bacterial transmission: results of multi-compartment models.* Bull Math Biol, 2007. **69**(7): p. 2227-48.

104. Borg, M.A., et al., *Correlation between meticillin-resistant Staphylococcus aureus prevalence and infection control initiatives within southern and eastern Mediterranean hospitals.* J Hosp Infect, 2009. **71**(1): p. 36-42.

105. Wendt, C., et al., *Prevalence of colonization with vancomycin-resistant enterococci in various population groups in Berlin, Germany.* J Hosp Infect. , 1999. **42**(3): p. 193-200.

106. Christiansen, K.J., et al., *Eradication of a Large Outbreak of a Single Strain of vanB Vancomycin‐Resistant Enterococcus faecium at a Major Australian Teaching Hospital.* Infect Control Hosp Epidemiol, 2004. **25**(5): p. 384-90.

107. Couderc, C., et al., *Fluoroquinolone use is a risk factor for methicillin-resistant Staphylococcus aureus acquisition in long-term care facilities: a nested case-case-control study.* Clin Infect Dis, 2014. **59**(2): p. 206-15.

108. Davenport, C., et al., *Decisional Guidance Tool for Antibiotic Prescribing in the Skilled Nursing Facility.* J Am Geriatr Soc, 2020. **68**(1): p. 55-61.

109. Domenech de Celles, M., et al., *Identifying more epidemic clones during a hospital outbreak of multidrug-resistant Acinetobacter baumannii.* PLoS One, 2012. **7**(9): p. e45758.

110. Emaneini, M., et al., *Nasal carriage rate of methicillin resistant Staphylococcus aureus among Iranian healthcare workers: a systematic review and meta-analysis.* Rev Soc Bras Med Trop, 2017. **50**(5): p. 590-597.

111. Fulchini, R., et al., *Antibiotic-resistant pathogens in different patient settings and identification of surveillance gaps in Switzerland - a systematic review.* Epidemiol Infect, 2019. **147**: p. e259.

112. Gaonkar, T.A., et al., *An alcohol hand rub containing a synergistic combination of an emollient and preservatives: prolonged activity against transient pathogens.* J Hosp Infect, 2005. **59**(1): p. 12-8.

113. Gorrie, C.L., et al., *Antimicrobial-Resistant Klebsiella pneumoniae Carriage and Infection in Specialized Geriatric Care Wards Linked to Acquisition in the Referring Hospital.* Clin Infect Dis, 2018. **67**(2): p. 161-170.

114. Hall, T.J., et al., *A comparison of the antibacterial efficacy and cytotoxicity to cultured human skin cells of 7 commercial hand rubs and Xgel, a new copper-based biocidal hand rub.* Am J Infect Control, 2009. **37**(4): p. 322-6.

115. Ho, H.J., et al., *Outbreak of New Delhi metallo-beta-lactamase-1-producing Enterobacter cloacae in an acute care hospital general ward in Singapore.* Am J Infect Control, 2016. **44**(2): p. 177-82.

116. Hughes, A., et al., *An outbreak of vanA vancomycin-resistant Enterococcus faecium in a hospital with endemic vanB VRE.* Infect Dis Health, 2019. **24**(2): p. 82-91.

117. Jimenez, A., et al., *Outbreak of Klebsiella pneumoniae Carbapenemase-Producing Citrobacter freundii at a Tertiary Acute Care Facility in Miami, Florida.* Infect Control Hosp Epidemiol, 2017. **38**(3): p. 320-326.

118. Kardas-Sloma, L., et al., *Universal or targeted approach to prevent the transmission of extended-spectrum beta-lactamase-producing Enterobacteriaceae in intensive care units: a cost-effectiveness analysis.* BMJ Open, 2017. **7**(11): p. e017402.

119. Knowles, S., et al., *An outbreak of multiply resistant Serratia marcescens: the importance of persistent carriage.* Bone Marrow Transplant, 2000. **25**(8): p. 873-7.

120. Kock, R., et al., *Persistence of nasal colonization with human pathogenic bacteria and associated antimicrobial resistance in the German general population.* New Microbes New Infect, 2016. **9**: p. 24-34.

121. Kypraios, T., et al., *Effect of systemic antibiotics and topical chlorhexidine on meticillin-resistant Staphylococcus aureus carriage in intensive care unit patients.* J Hosp Infect, 2011. **79**(3): p. 222-6.

122. Larsson, A.K., et al., *Duration of methicillin-resistant Staphylococcus aureus colonization after diagnosis: a four-year experience from southern Sweden.* Scand J Infect Dis, 2011. **43**(6-7): p. 456-62.

123. Lietzau, S., et al., *Household contacts were key factor for children's colonization with resistant Escherichia coli in community setting.* J Clin Epidemiol, 2007. **60**(11): p. 1149-55.

124. Lu, P.L., et al., *Risk factors and molecular analysis of community methicillin-resistant Staphylococcus aureus carriage.* J Clin Microbiol, 2005. **43**(1): p. 132-9.

125. Ma, X., et al., *First multicenter study on multidrug resistant bacteria carriage in Chinese ICUs.* BMC Infect Dis, 2015. **15**: p. 358.

126. Masadeh, M.M., et al., *Identification, characterization and antibiotic resistance of bacterial isolates obtained from waterpipe device hoses.* Int J Environ Res Public Health, 2015. **12**(5): p. 5108-15.

127. Mills, J.P., et al., *The devil is in the details: Factors influencing hand hygiene adherence and contamination with antibiotic-resistant organisms among healthcare providers in nursing facilities.* Infect Control Hosp Epidemiol, 2019. **40**(12): p. 1394-1399.

128. Mirhoseini, S.H., et al., *Hospital air: A potential route for transmission of infections caused by beta-lactam-resistant bacteria.* Am J Infect Control, 2016. **44**(8): p. 898-904.

129. Munoz-Price, L.S., *Long-term acute care hospitals.* Clin Infect Dis, 2009. **49**(3): p. 438-43.

130. Murray, C.K., et al., *Recovery of Multidrug-Resistant Bacteria From Combat Personnel Evacuated From Iraq and Afghanistan at a Single Military Treatment Facility.* 2009.

131. Murray, R.J., et al., *Outbreak of invasive methicillin-resistant Staphylococcus aureus infection associated with acupuncture and joint injection.* Infect Control Hosp Epidemiol, 2008. **29**(9): p. 859-65.

132. Murthy, A., et al., *Cost-effectiveness of universal MRSA screening on admission to surgery.* Clinical Microbiology and Infection, 2010. **16**(12): p. 1747–53.

133. Muthukrishnan, G., et al., *Exoproteome of Staphylococcus aureus reveals putative determinants of nasal carriage.* J Proteome Res, 2011. **10**(4): p. 2064-78.

134. Nagao, M., et al., *Control of an outbreak of carbapenem-resistant Pseudomonas aeruginosa in a haemato-oncology unit.* J Hosp Infect, 2011. **79**(1): p. 49-53.

135. Pourramezan, N., S. Ohadian Moghadam, and M.R. Pourmand, *Methicillin-resistant Staphylococcus aureus tracking spread among health-care workers and hospitalized patients in critical wards at a university hospital, Tehran, Iran.* New Microbes New Infect, 2019. **27**: p. 29-35.

136. Roghmann, M.C., et al., *Comparison of the Microbiota of Older Adults Living in Nursing Homes and the Community.* mSphere, 2017. **2**(5).

137. Ruscher, C., et al., *Inguinal skin colonization with multidrug-resistant bacteria among residents of elderly care facilities: frequency, persistence, molecular analysis and clinical impact.* Int J Med Microbiol, 2014. **304**(8): p. 1123-34.

138. Schwaber, M.J. and Y. Carmeli, *An ongoing national intervention to contain the spread of carbapenem-resistant enterobacteriaceae.* Clin Infect Dis, 2013. **58**(5): p. 697-703.

139. Seekatz, A.M., et al., *Gut microbiota and clinical features distinguish colonization with Klebsiella pneumoniae carbapenemase-producing Klebsiella pneumoniae at the time of admission to a long-term acute care hospital.* Open Forum Infect Dis, 2018. **5**(8): p. ofy190.

140. Shiferaw, T., et al., *Bacterial contamination, bacterial profile and antimicrobial susceptibility pattern of isolates from stethoscopes at Jimma University Specialized Hospital.* Ann Clin Microbiol Antimicrob, 2013. **12**(39).

141. Solberg, C.O., *Spread of Staphylococcus aureus in Hospitals: Causes and Prevention.* Scand J Infect Dis, 2000. **32**(6): p. 587-95.

142. Taori, S.K., et al., *Candida auris outbreak: Mortality, interventions and cost of sustaining control.* J Infect, 2019. **79**(6): p. 601-611.

143. Tosas Auguet, O., et al., *Frequent Undetected Ward-Based Methicillin-Resistant Staphylococcus aureus Transmission Linked to Patient Sharing Between Hospitals.* Clin Infect Dis, 2018. **66**(6): p. 840-848.

144. van Balen, J., et al., *Understanding the introduction and circulation of environmental methicillin-resistant Staphylococcus aureus in a large academic medical center during a nonoutbreak, year-long period.* Am J Infect Control, 2016. **44**(8): p. 925-30.

145. van der Donk, C.F., et al., *Prevalence and spread of multidrug resistant Escherichia coli isolates among nursing home residents in the southern part of The Netherlands.* J Am Med Dir Assoc, 2013. **14**(3): p. 199-203.

146. van der Zwet, W.C., et al., *Nosocomial outbreak of gentamicinresistant Klebsiella pneumoniae in a neonatal intensive care unit controlled by a change in antibiotic policy.* J Hosp Infect, 1999. **42**(4): p. 295-302.

147. Warde, E., E. Davies, and A. Ward, *Control of a multidrug-resistant Acinetobacter baumannii outbreak.* Br J Nurs, 2019. **28**(4): p. 242-8.

148. Welsh, R.M., et al., *Survival, Persistence, and Isolation of the Emerging Multidrug-Resistant Pathogenic Yeast Candida auris on a Plastic Health Care Surface.* J Clin Microbiol, 2017. **55**(10): p. 2996-3005.

149. Zimmerman, F.S., et al., *Duration of carriage of carbapenem-resistant Enterobacteriaceae following hospital discharge.* Am J Infect Control, 2013. **41**(3): p. 190-4.

150. Zollner-Schwetz, I., et al., *Colonization of long term care facility patients with MDR-Gram-negatives during an Acinetobacter baumannii outbreak.* Antimicrob Resist Infect Control, 2017. **6**: p. 49.

# Data Visualization Assessment Form.

Assessor

o Assessor 1

o Assessor 2

Data visualization ID

▼ ID1 … ID180

Year article

________________________________________________________________

First impression

o 1: poor

o 2

o 3

o 4

o 5: good

Viz type

▢ Violin

▢ Density plot

▢ Boxplot

▢ Histogram

▢ Scatter plot

▢ Connected scatter plot

▢ Bubble plot

▢ Area plot

▢ Stacked area plot

▢ Stream plot

▢ Line chart

▢ Ridge line

▢ Correlogram

▢ Heatmap

▢ Dendrogram

▢ Bar chart

▢ Stacked bar chart

▢ Lollipop chart

▢ Doughnut chart

▢ Treemap

▢ Circular packaging plot

▢ Venn diagram

▢ Sunburst diagram

▢ Spider plot

▢ Sankey diagram

▢ Network plot

▢ Chord diagram

▢ Arc diagram

▢ Hive plot

▢ Hierarchical edge bundling

▢ Other_______________

▢ Combined

Faceting (multiple groups in a grid)

o Yes

o No

Actions

▢ Analyze

▢ Search

▢ Query - Identify

▢ Query - Compare

▢ Query - Summarize

Attributes

o 1

o 2

o 3

o 4

o 5

o 6

Attribute names and types

|  | Attribute name | Attribute type | | | | |
| --- | --- | --- | --- | --- | --- | --- |
|  | Name | Categorical | Ordinal | Quantitative | NA | Mismatch |
| Attr. 1 | ▢ | ▢ | ▢ | ▢ | ▢ | ▢ |
| Attr. 2 | ▢ | ▢ | ▢ | ▢ | ▢ | ▢ |
| Attr. 3 | ▢ | ▢ | ▢ | ▢ | ▢ | ▢ |
| Attr. 4 | ▢ | ▢ | ▢ | ▢ | ▢ | ▢ |
| Attr. 5 | ▢ | ▢ | ▢ | ▢ | ▢ | ▢ |
| Attr. 6 | ▢ | ▢ | ▢ | ▢ | ▢ | ▢ |

Marks

|  | Points | Lines | Areas | NA | Mismatch |
| --- | --- | --- | --- | --- | --- |
| Attr. 1 | ▢ | ▢ | ▢ | ▢ | ▢ |
| Attr. 2 | ▢ | ▢ | ▢ | ▢ | ▢ |
| Attr. 3 | ▢ | ▢ | ▢ | ▢ | ▢ |
| Attr. 4 | ▢ | ▢ | ▢ | ▢ | ▢ |
| Attr. 5 | ▢ | ▢ | ▢ | ▢ | ▢ |
| Attr. 6 | ▢ | ▢ | ▢ | ▢ | ▢ |

Channels / position

|  | Horizontal | Vertical | Both | NA | Mismatch |
| --- | --- | --- | --- | --- | --- |
| Attr. 1 | ▢ | ▢ | ▢ | ▢ | ▢ |
| Attr. 2 | ▢ | ▢ | ▢ | ▢ | ▢ |
| Attr. 3 | ▢ | ▢ | ▢ | ▢ | ▢ |
| Attr. 4 | ▢ | ▢ | ▢ | ▢ | ▢ |
| Attr. 5 | ▢ | ▢ | ▢ | ▢ | ▢ |
| Attr. 6 | ▢ | ▢ | ▢ | ▢ | ▢ |

Channels / colour - shape – tilt

|  | Color | Shape | Tilt | NA | Mismatch |
| --- | --- | --- | --- | --- | --- |
| Attr. 1 | ▢ | ▢ | ▢ | ▢ | ▢ |
| Attr. 2 | ▢ | ▢ | ▢ | ▢ | ▢ |
| Attr. 3 | ▢ | ▢ | ▢ | ▢ | ▢ |
| Attr. 4 | ▢ | ▢ | ▢ | ▢ | ▢ |
| Attr. 5 | ▢ | ▢ | ▢ | ▢ | ▢ |
| Attr. 6 | ▢ | ▢ | ▢ | ▢ | ▢ |

Channels / size

|  | Length | Area | Volume | NA | Mismatch |
| --- | --- | --- | --- | --- | --- |
| Attr. 1 | ▢ | ▢ | ▢ | ▢ | ▢ |
| Attr. 2 | ▢ | ▢ | ▢ | ▢ | ▢ |
| Attr. 3 | ▢ | ▢ | ▢ | ▢ | ▢ |
| Attr. 4 | ▢ | ▢ | ▢ | ▢ | ▢ |
| Attr. 5 | ▢ | ▢ | ▢ | ▢ | ▢ |
| Attr. 6 | ▢ | ▢ | ▢ | ▢ | ▢ |

Channels effectiveness for ordered attributes

|  | Position on common scale | Position on unaligned scale | Length (1D size) | Tilt/angle | Area (2D size) | Depth (3D position) | Color luminance / Color saturation | Curvature / Volume (3D size) | NA | Mismatch |
| --- | --- | --- | --- | --- | --- | --- | --- | --- | --- | --- |
| Attr. 1 | ▢ | ▢ | ▢ | ▢ | ▢ | ▢ | ▢ | ▢ | ▢ | ▢ |
| Attr. 2 | ▢ | ▢ | ▢ | ▢ | ▢ | ▢ | ▢ | ▢ | ▢ | ▢ |
| Attr. 3 | ▢ | ▢ | ▢ | ▢ | ▢ | ▢ | ▢ | ▢ | ▢ | ▢ |
| Attr. 4 | ▢ | ▢ | ▢ | ▢ | ▢ | ▢ | ▢ | ▢ | ▢ | ▢ |
| Attr. 5 | ▢ | ▢ | ▢ | ▢ | ▢ | ▢ | ▢ | ▢ | ▢ | ▢ |
| Attr. 6 | ▢ | ▢ | ▢ | ▢ | ▢ | ▢ | ▢ | ▢ | ▢ | ▢ |

Channels effectiveness for categorical attributes:

|  | Spatial position | Color hue | Motion | Shape | NA | Mismatch |
| --- | --- | --- | --- | --- | --- | --- |
| Attr. 1 | ▢ | ▢ | ▢ | ▢ | ▢ | ▢ |
| Attr. 2 | ▢ | ▢ | ▢ | ▢ | ▢ | ▢ |
| Attr. 3 | ▢ | ▢ | ▢ | ▢ | ▢ | ▢ |
| Attr. 4 | ▢ | ▢ | ▢ | ▢ | ▢ | ▢ |
| Attr. 5 | ▢ | ▢ | ▢ | ▢ | ▢ | ▢ |
| Attr. 6 | ▢ | ▢ | ▢ | ▢ | ▢ | ▢ |

Describe what is visualized:

________________________________________________________________

Interpretability without text

o 1 Not at all

o 2 Partially

o 3 Yes

Choice of plot type

o 1: poor

o 2

o 3

o 4

o 5: good

What can be improved?

________________________________________________________________

________________________________________________________________

________________________________________________________________

________________________________________________________________

________________________________________________________________

Remarks (e.g. chart junk)

________________________________________________________________

________________________________________________________________

________________________________________________________________

________________________________________________________________

________________________________________________________________

Last impression

o 1: poor

o 2

o 3

o 4

o 5: good

# Visualization types per attribute.

| **Table S2** | | | | | | | | | | | | | | |
| --- | --- | --- | --- | --- | --- | --- | --- | --- | --- | --- | --- | --- | --- | --- |
| **Attribute** | **Line chart** | **Bar chart** | **Stacked bar chart** | **Dendrogram** | **Forest plot** | **Range plot** | **Bubble plot** | **Pie chart** | **Tornado plot** | **Scatter plot** | **Bma image plot** | **Heatmap** | **Stacked area plot** | **Correlogram** |
| Antimicrobial | 31.03% | **34.48%** | 24.14% |  |  |  |  | 6.90% |  |  | 3.45% |  |  |  |
| Antimicrobial consumption | **46.15%** | 23.08% | 25.64% |  |  |  |  | 2.56% |  | 2.56% |  |  |  |  |
| Bacteria | 8.33% | **58.33%** | 8.33% |  |  |  | 8.33% |  |  |  |  | 8.33% | 8.33% |  |
| Case |  |  | 16.67% | 16.67% |  | **66.67%** |  |  |  |  |  |  |  |  |
| Confidence Interval | 18.18% | 27.27% |  |  | **45.45%** |  |  |  |  | 9.09% |  |  |  |  |
| Cohort | 20.00% | **40.00%** | 20.00% |  |  | 20.00% |  |  |  |  |  |  |  |  |
| Compliance | 11.11% | **77.78%** | 11.11% |  |  |  |  |  |  |  |  |  |  |  |
| Cost | **40.00%** |  |  |  |  |  |  |  | **40.00%** | 20.00% |  |  |  |  |
| Count | 14.81% | **33.33%** | 18.52% |  | 3.70% | 7.41% | 14.81% |  |  | 7.41% |  |  |  |  |
| Cut-off | **30.77%** | 15.38% |  | 15.38% | 15.38% |  | 7.69% |  | 7.69% | 7.69% |  |  |  |  |
| Diagnosis |  | **44.44%** | 33.33% |  |  |  | 11.11% | 11.11% |  |  |  |  |  |  |
| Error |  | **80.00%** |  |  |  | 20.00% |  |  |  |  |  |  |  |  |
| Event | **25.00%** | **25.00%** | **25.00%** |  | 8.33% | 16.67% |  |  |  |  |  |  |  |  |
| Incidence | **37.04%** | 22.22% | 22.22% |  | 3.70% | 7.41% |  |  |  | 3.70% |  |  | 3.70% |  |
| Numeric value | **47.83%** | 26.09% |  |  | 13.04% | 4.35% |  |  |  | 4.35% | 4.35% |  |  |  |
| Percentage | 7.41% | **51.85%** | 22.22% |  | 3.70% |  |  | 11.11% |  |  |  | 3.70% |  |  |
| Regression | **62.50%** |  |  |  |  |  | 12.50% |  |  | 25.00% |  |  |  |  |
| Resistance | 35.14% | **40.54%** | 10.81% |  | 2.70% |  | 5.41% |  |  | 5.41% |  |  |  |  |
| Sample |  | 8.33% | 8.33% | **50.00%** |  | 16.67% |  |  |  |  |  | 8.33% |  | 8.33% |
| Setting | 15.22% | **41.30%** | 19.57% | 2.17% | 8.70% | 4.35% |  | 2.17% |  | 4.35% |  | 2.17% |  |  |
| Similarity |  |  |  | **80.00%** |  |  |  |  |  |  |  | 10.00% |  | 10.00% |
| Statistics | **33.33%** | **33.33%** |  |  | 16.67% |  |  |  | 8.33% |  |  | 8.33% |  |  |
| Survey answer |  | **50.00%** | 37.50% |  |  |  | 12.50% |  |  |  |  |  |  |  |
| Time | **52.63%** | 25.00% | 10.53% |  |  | 6.58% | 2.63% |  |  | 1.32% |  |  | 1.32% |  |

# Visual characteristics per attribute.

| **Table S3** | | | | | | | | | | | | | | | |
| --- | --- | --- | --- | --- | --- | --- | --- | --- | --- | --- | --- | --- | --- | --- | --- |
| Attribute | Channel - position | | |  | Channel - marks | | |  | Channel - colour/shape/tilt | | |  | Channel - size | | |
|  | Horizontal | Vertical | Both |  | Areas | Lines | Points |  | Color | Shape | Tilt |  | Area | Length | Volume |
| Antimicrobial | **72.73%** | 27.27% |  |  |  |  |  |  | **64.71%** | 35.29% |  |  |  |  |  |
| Antimicrobial consumption | 3.13% | **96.88%** |  |  | 3.03% | **48.48%** | **48.48%** |  | 33.33% | **66.67%** |  |  | 6.25% | **93.75%** |  |
| Bacteria | **71.43%** | 14.29% | 14.29% |  |  |  |  |  | **80.00%** | 20.00% |  |  |  |  |  |
| Case | 20.00% | **80.00%** |  |  |  |  |  |  |  |  |  |  |  |  |  |
| Confidence interval | **60.00%** | 30.00% | 10.00% |  |  | **81.82%** | 18.18% |  |  | **100.00%** |  |  |  | **100.00%** |  |
| Cohort | 40.00% | **60.00%** |  |  |  |  | **100.00%** |  | **75.00%** | 25.00% |  |  |  |  |  |
| Compliance |  | **100.00%** |  |  |  | **83.33%** | 16.67% |  | **100.00%** |  |  |  |  | **100.00%** |  |
| Cost | 40.00% | **60.00%** |  |  |  | **50.00%** | **50.00%** |  |  |  |  |  |  | **100.00%** |  |
| Count | 23.81% | **76.19%** |  |  | 5.26% | **63.16%** | 31.58% |  | **100.00%** |  |  |  | 18.75% | **75.00%** | 6.25% |
| Cut-off | **66.67%** | 11.11% | 22.22% |  | 12.50% | **75.00%** | 12.50% |  | **100.00%** |  |  |  | **50.00%** | **50.00%** |  |
| Diagnosis | **80.00%** | 20.00% |  |  |  |  |  |  | **75.00%** | 25.00% |  |  |  |  |  |
| Error | 40.00% | **60.00%** |  |  |  | **100.00%** |  |  | **100.00%** |  |  |  |  | **100.00%** |  |
| Event | **100.00%** |  |  |  |  | 33.33% | **66.67%** |  | 25.00% | **75.00%** |  |  |  | **100.00%** |  |
| Incidence | 8.70% | **91.30%** |  |  | 5.00% | 45.00% | **50.00%** |  | 28.57% | **71.43%** |  |  | 9.09% | **90.91%** |  |
| Numeric value | **55.00%** | 40.00% | 5.00% |  | 6.67% | 40.00% | **53.33%** |  |  |  |  |  | 33.33% | **66.67%** |  |
| Percentage | 34.78% | **60.87%** | 4.35% |  | 12.50% | **75.00%** | 12.50% |  | **66.67%** | 33.33% |  |  | 8.70% | **78.26%** | 13.04% |
| Regression |  | **71.43%** | 28.57% |  |  | **71.43%** | 28.57% |  | 33.33% | **66.67%** |  |  |  |  |  |
| Resistance | 7.41% | **92.59%** |  |  | 3.70% | **51.85%** | 44.44% |  | **77.78%** | 22.22% |  |  | 7.14% | **92.86%** |  |
| Sample |  | **100.00%** |  |  |  |  |  |  | **100.00%** |  |  |  |  |  |  |
| Setting | **60.87%** | 39.13% |  |  |  | 33.33% | **66.67%** |  | **59.38%** | 40.63% |  |  |  |  |  |
| Similarity | **100.00%** |  |  |  |  | **100.00%** |  |  |  |  |  |  |  | **100.00%** |  |
| Statistics | 22.22% | **77.78%** |  |  |  | **50.00%** | **50.00%** |  | **66.67%** | 33.33% |  |  |  | **100.00%** |  |
| Survey answer | 40.00% | **60.00%** |  |  |  |  | **100.00%** |  | **75.00%** | 25.00% |  |  |  |  |  |
| Time | **100.00%** |  |  |  |  | **100.00%** |  |  | **55.56%** | 33.33% | 11.11% |  |  | **100.00%** |  |

# Channel effectiveness for quantitative, ordinal and categorical attributes.

| **Table S4** | | | |
| --- | --- | --- | --- |
| Effectiveness | Quantitative | Ordinal | Categorical |
| Position on common scale | **82.8%** | **83.0%** |  |
| Position on unaligned scale | 1.1% | 1.0% |  |
| Length (1D size) | 3.4% | 2.0% |  |
| Area (2D size) | 6.7% | 2.0% |  |
| Depth (3D position) | 0.4% |  |  |
| Color luminance/  Color saturation | 5.6% | 12.0% |  |
| Spatial position |  |  | 1.3% |
| Color hue |  |  | **68.9%** |
| Shape |  |  | 29.8% |

# Problems and illustrative quotes.

| **Table S5.** | | | |
| --- | --- | --- | --- |
| Code (axial) | Code (open) | Illustrative quotes | Count |
| Missing labels, annotations, legend and/or abbreviation explanations | Legend/caption is missing/unclear | Legend is missing. | 26 |
|  | Labels for lines/points are missing/unclear | Line is not labeled and it is unclear what it represents. | 23 |
|  | Labels for axes are missing/unclear | Y-axis label is missing. | 20 |
|  | Annotation/direct labeling overflow | Difficult to read which sites are statistically significantly different. | 14 |
|  | Abbreviations not explained | Abbreviations not explained. | 12 |
|  | Use of colours not explained | Not clear what colours mean in visualization. | 7 |
| Subtotal | | | 102 |
| Axes not readable | Axis intervals uneven (within visualization and between faceted visualizations) | Plots use different y-axis, so it is difficult to compare them. | 17 |
|  | Axes texts not clearly readable | Too much text in y-axis labels. | 11 |
|  | Too short/dense axes/intervals | Lines are cut above 100. | 5 |
|  | Uneven bar placement | White separation lines not shown for each bar. | 1 |
|  | Axis intervals unlogical (within visualization and between faceted visualizations) | X-axis intervals are not easy to read/compare. | 1 |
| Subtotal | | | 35 |
| Colour scheme mismatch | Groups not distinguishable by colours | Use of similar colours, so hard to distinguish. | 14 |
|  | Non-intuitive colour schemes used | Counter-intuitive coding (colour luminance levels for min max should be switched). | 6 |
|  | Categorical colours used for ordered attribute | Mismatch of categorical colours for order attribute. | 5 |
|  | Groups not distinguishable from background | Colour for one group should not be the same as background (white). | 2 |
| Subtotal | | | 27 |
| Hidden data points by overlaps | Overlap of shapes problematic | Points could be double, but not differentiable because of overlap. | 7 |
| Subtotal | | | 7 |
| Using suboptimal channel effectiveness | Groups not distinguishable by shapes | Shapes are very difficult to differentiate. | 12 |
|  | Sub-effective channel is chosen | Shape/annotation not most effective in distinguishing between colonized/infected (colour higher effectiveness) | 3 |
| Subtotal | | | 15 |
| Size scale indistinguishable | Differences in size not clear | Standard errors difficult to read (too small). | 10 |
|  | Groups not distinguishable by shape size | Difference in study weight (represented with square size) not readable. | 3 |
|  | Contrasts between groups not clear | Difficult to follow one patient line. | 2 |
| Subtotal | | | 15 |
| Missing channel | Line types not used to distinguish between groups | Different line types could improve distinguishing the different groups. | 2 |
|  | Colors not used to compare between visualization/groups | Colours could have been used to make comparison of isolates across groups easier. | 2 |
| Subtotal | | | 4 |
| Visualization type does not (optimally) fit data | Other visualization type preferred | Too many categories for the plot type (e.g. bar chart) would be better). | 21 |
| Subtotal | | | 21 |
| Data points/lines on double axes | Double Y-axes difficult to read | Double y-axis confuse (not easy to apprehend which line is on which axis). | 11 |
| Subtotal | | | 11 |
| Channel overflow | Double use of shape and colour | Unnecessary use of two channels for one attribute. | 8 |
|  | Unnecessary use of shape sizes | Unequal size of point marks (without meaning). | 1 |
|  | Unnecessary use of colour | Unnecessary colour channel for intervention period. | 1 |
|  | Too many colours | Too many colours. | 1 |
| Subtotal | | | 11 |
| Attribute overflow | Too many attributes | Plot is overloaded with attributes. | 2 |
|  | Relating attributes that are not related | Stacked bar chart used for unrelated attributes (stacking does not make sense) | 1 |
| Subtotal | | | 3 |
| Information sparsity | Could be text | Almost too little information. | 1 |
| Subtotal | | | 1 |
| Incoherent ordering | Data not ordered coherently | Colonized/infected not consequently ordered in bars. | 1 |
| Subtotal | | | 1 |
| Grand Total | | | 253 |
